# Air-Raids and Bombardment of Institutions

**Published:** 1915-08-21

**Authors:** 


### AIR-RAIDS AND BOMBARDMENT OF INSTITUTIONS.

### The Government Insurance Scheme.

Whatever may be the view of private individuals as to the necessity or advisability of insurance against the risk of damage by air raids, many of those responsible for the buildings and contents of the hospitals throughout the country participated cheerfully in the Government scheme. Hospital managers have always considered it a matter of prudence to insure against the risk of fire, and now that the country—or at least certain districts—is faced with a new danger which was not covered by the existing fire insurance policies it was necessary to consider whether prudence did not dictate the extension of the principle of insurance to cover the new risk. In this matter there is no precedent. The conditions are entirely new, and it is necessary to face the facts as they are. From the moment that the Government scheme was adopted the Government was relieved of all liability in respect of damage arising through air raids or from bombardment from enemy guns not landed in this country, unless the property damaged was insured under the Government scheme. There is, therefore, no possibility of gaining compensation from the State in the event of damage arising from either cause unless advantage has been taken of the insurance system provided by the Government.

The Government is making use of approved fire insurance offices as agents for collecting the premiums, issuing the policies, and settling the claims, but it is necessary to point out that the offices are only agents in the matter, and persons—or corporations—insuring under the scheme have the full security of the State on which they may rely in the event of it being necessary to make a claim.

### The Two Policies.

The policies, moreover, are simple in form, and their conditions are such that but few exceptions can be taken to them. It should be noted particularly that there are two policies. One policy covers the risk of the property insured, or any part thereof, "being destroyed or damaged directly or indirectly by aerial craft (hostile or otherwise) or shots, shells, bombs, or missiles from or used against aerial craft." The other policy, besides covering this risk, also provides indemnity against destruction or damage arising by "bombardment by hostile guns not landed on British territory."

Thus it will be seen that those who own property at or near the coast are provided with the means of covering the additional risk to which they are liable as compared with those whose property is beyond the range of hostile naval guns. The cost of the insurance is small in comparison with the amount of the loss which would have to be made good in the event of the property being damaged or destroyed. For hospitals the premium required is at the rate of 2s. for each £100 insured, such premium covering the air-raid risk for twelve months. In view of the fact that air raids have been not infrequent of late, it seems desirable for prudent trustees who have not already done so to lose no time in effecting the insurance.

